# Prognostic significance of subclassification of stage IIB lung cancer: a retrospective study of 226 patients

**DOI:** 10.18632/oncotarget.17405

**Published:** 2017-04-25

**Authors:** Nanchang Yin, Minwen Ha, Yu Liu, Huizi Gu, Zetian Zhang, Wei Liu

**Affiliations:** ^1^ Department of Thoracic Surgery, The First Affiliated Hospital of Jinzhou Medical University, Jinzhou 121001, China; ^2^ Department of Medical Oncology, The First Affiliated Hospital of Jinzhou Medical University, Jinzhou 121001, China; ^3^ Department of Thoracic Surgery, Liaoning Cancer Hospital and Institute, Shenyang 110042, China; ^4^ Department of Internal Neurology, The Second Hospital of Dalian Medical University, Dalian 116027, China; ^5^ Shenyang Yike Biotechnology Co., Ltd, Shenyang 110000, China

**Keywords:** lung cancer, lymph node metastasis, T category, prognosis, stage IIB

## Abstract

We investigated the prognostic significance of subclassification of stage IIB lung cancer according to the eighth tumor-node-metastasis (TNM) classification. To this purpose, the prognostic outcomes of 226 stage IIB lung cancer patients who underwent surgery without adjuvant therapies between 2001 and 2010 were evaluated retrospectively based on the eighth TNM classification. Of the 226 patients, 23, 30, 118 and 55 had pT1b, pT1c, pT2a, and pT2b stage cancers, respectively. Their 5-year survival rates were 67%, 33%, 21%, and 27%, respectively. There was no significant difference in the 5-year survival between T1b and T1c, between T1c and T2a, and between T2a and T2b (p = 0.128, 0.105, and 0.403, respectively). There were significant differences in the 5-year survival between T1b and T2a, between T1b and T2b, and between T1c and T2b (p = 0.005, 0.002, and 0.042, respectively). The 5-year survival of patients with pleural invasion and vessel invasion was significantly worse than that of their counterparts (p = 0.009 and <0.001, respectively). Subclassification of stage IIB lung cancer is of prominent prognostic significance. It is recommended that the current stage be subclassified, in order to more accurately predict the prognosis of patients.

## INTRODUCTION

Lung cancer is the major cause of cancer death in China [1] and worldwide [2]. Classification of lung cancer is important to establish therapeutic strategies and predict prognosis [3]. Early detection and the introduction of rational lymphadenectomy and several other therapeutic modalities have improved the survival of patients with lung cancer [4–5]. In particular, advances in high-throughput sequencing technologies and targeted therapies have improved therapeutic efficacy and long-term prognosis [6–8]. However, currently, limited by China's economic level, many patients with lung cancer only undergo radical surgery, and do not receive other adjuvant treatments such as chemotherapy, radiotherapy, and targeted drug therapies [9]. The postoperative survival of these patients can to a large extent reflect the natural course of the disease after radical surgery for lung cancer. We systematically analyzed and followed up lung cancer patients who received radical surgery at the Department of Thoracic Surgery in our hospital and other hospitals in the past ten years. Patients who did not receive any adjuvant therapies were selected and their survival was analyzed, to determine the survival of patients with different stages of lung cancer.

Tumor size and lymph node status are important prognostic factors for lung cancer [10–12]. In the past, we have used Union for International Cancer Control (UICC) seventh edition of the tumor-node-metastasis (TNM) staging criteria to guide clinical practice [13]. In the eighth edition of TNM staging, the criteria for T staging have been revised, but those for N and M staging remained largely unchanged [14–22]. With the change in T staging criteria, the corresponding TNM staging has also changed. The criteria for T staging are very detailed, and the size difference between stages is 1 cm in almost all cases. Therefore, whether classifying patients with different T staging into the same pathological stage can truly reflect patient prognosis needs to be reconsidered. We first looked at stage IIB in the eighth edition of TNM staging for lung cancer. In our study, there were no patients at the T3N0M0 or T1aN1M0 stage; therefore, according to T staging, the patients were divided into four groups: T1b, T1c, T2a, and T2b. According to the seventh edition of TNM staging, the four groups correspond to the three T stages of T1a, T1b, and T2a. Patients with N1 stage are classified into the IIA stage.

We observed the prognosis of patients in groups T1b, T1c, T2a, and T2b with N1 stage, who received only radical surgery, thus providing a reference for future development of TNM staging criteria that are more detailed and closer to the actual course of lung cancer, as well as for precise treatments of lung cancer.

## RESULTS

### Clinicopathological characteristics of stage IIB lung cancer patients

A total of 226 patients were included in this study. Of those, 66% were younger than 60 years old, and 57% were female. Of the 226 patients, 23, 30, 118 and 55 had pT1b, pT1c, pT2a, and pT2b stage cancers, respectively. T1b stage corresponds to the T1a stage of the seventh edition of TNM staging, while T1c stage corresponds to the T1b stage in the seventh edition. Therefore, the number of patients and their proportion were the same in the two staging criteria. The T2a stage in the seventh edition is divided into T2a and T2b stages in the eighth edition, and T2a patients accounted for a larger proportion, which was equal to the 2/3 of the T2a patients classified according to the original seventh edition criteria. In the histological classification, the patients of medium grade accounted for more than 50%, while the high-grade patients accounted for more than 1/3. As for the pathological types, adenocarcinoma accounted for nearly 2/3 of the patients, patients with the tumor invasion of the pleura accounted for nearly 2/3, and patients with invasion of blood vessels accounted for slightly higher than 1/3 (Table 1).

**Table 1:** Overall survival according to demographic and clinical characteristics of stage IIB patients (n = 226)

Characteristics	n (%)	5-year survival (%)	p value
**Age**			0.799
<60 years	150 (66)	28	
≥60 years	76 (34)	37	
**Sex**			0.654
Women	130 (57)	33	
Men	96 (43)	33	
**Seventh pathological tumor stage**			0.003
T1a	23 (10)	67	
T1b	30 (13)	33	
T2a	173 (77)	25	
**Eighth pathological tumor stage**			0.005
T1b	23 (10)	67	
T1c	30 (13)	33	
T2a	118 (52)	21	
T2b	55 (25)	27	
**Grade**			0.346
Low	16 (7)	13	
Intermediate	124 (55)	42	
High	86 (38)	25	
**Histological type**			0.151
Squamous	89 (39)	31	
Adenocarcinoma	137 (61)	34	
**Pleural invasion**			0.009
No	89 (39)	42	
Yes	137 (61)	26	
**Vessel invasion**			<0.001
No	141 (62)	42	
Yes	85 (38)	16	

### Survival according to the subclassification of stage IIB lung cancer in the eighth TNM classification

**Figure 1:**
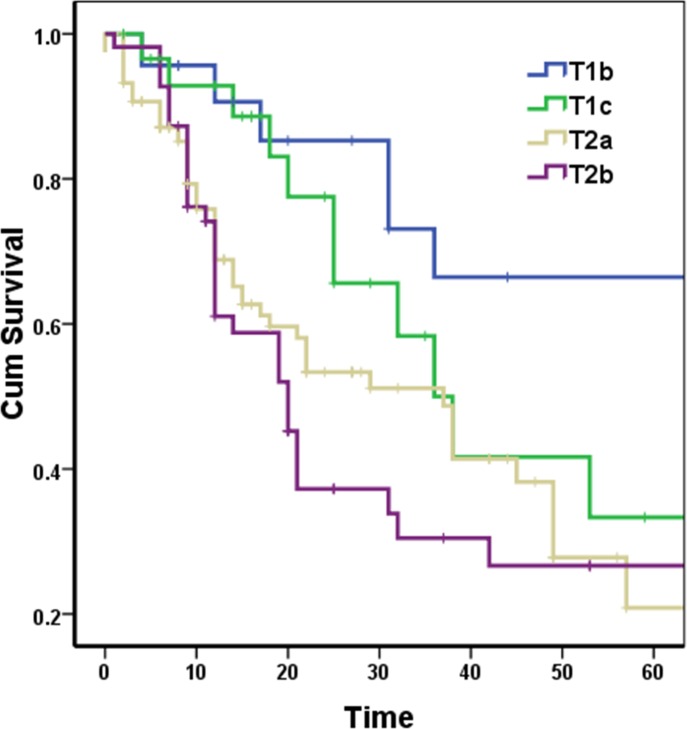
Survival curves according to four T stages in stage IIB lung cancer patients

Patients were followed up for at least 63 months, with a medium of 57 months. The 5-year survival rate of all patients with stage IIB cancer was 32.4%. There were significant differences in overall survival among the four T stages (Figure 1; p = 0.005). The 5-year survival rates of the four T stages were 67%, 33%, 21%, and 27%, respectively (Table 1).

**Figure 2:**
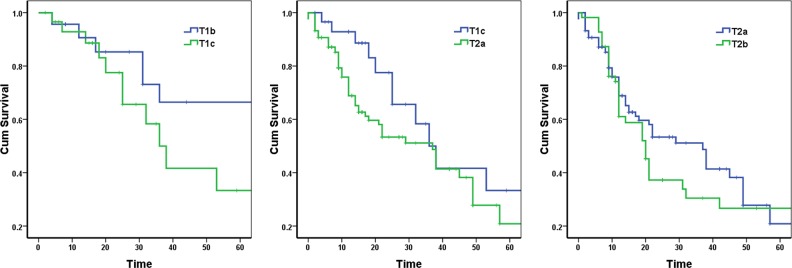
Survival curves according to two neighboring T stages in stage IIB lung cancer patients

There was no significant difference of the 5-year survival between T1b and T1c, between T1c and T2a, and between T2a and T2b (p = 0.128, 0.105, and 0.403, respectively) (Figure 2).

**Figure 3:**
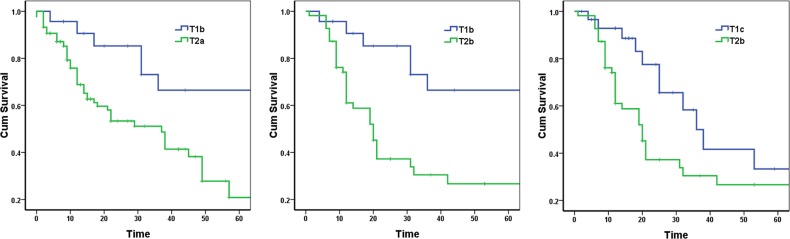
Survival curves according to two non-neighboring T stages in stage IIB lung cancer patients

However, there were significant differences of the 5-year survival between T1b and T2a, between T1b and T2b, and between T1c and T2b (p = 0.005, 0.002, and 0.042, respectively) (Figure 3).

**Figure 4:**
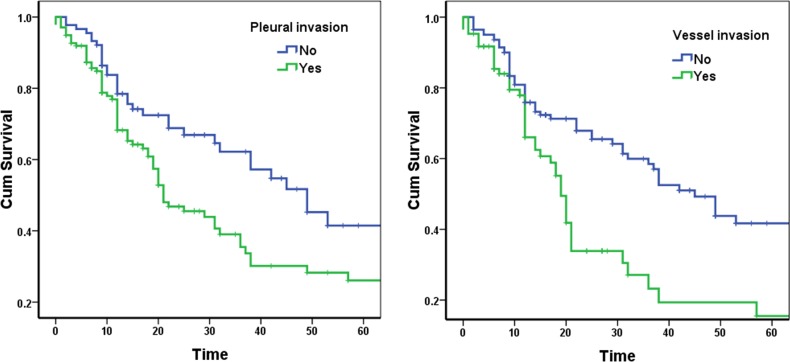
Survival curves according to pleural invasion and vessel invasion in stage IIB lung cancer patients

In addition, the 5-year survival of patients with pleural invasion and vessel invasion was significantly worse than that of their counterparts (p = 0.009 and <0.001, respectively) (Table 1, Figure 4).

## DISCUSSION

In this study, patients with stage IIB lung cancer who only underwent radical surgery were selected. Since only a limited proportion of patients can receive radical surgery, and stage IIB is a relatively late stage in these patients, the distribution of clinical and pathological features of these patients is different from that reported in other similar studies [9, 23]. A large proportion of the patients were younger than 60 years old, which may be explained by the fact that tumors in older patients are often at an even later stage. Meanwhile, the proportion of female patients was slightly higher than that of men, which may due to the fact that the disease in female patients is often at an earlier stage than in male patients. Regardless of the differences in age or gender distribution, the prognosis of patients was not affected significantly. Similar to previous studies, for the distribution of histological grades, most cases were mid–high grade, and low-grade tumors accounted for only a very small percentage. There was no significant difference in survival between the three histological grades, which may be due to the small number of patients with low-grade tumors and the presence of biased survival data. Adenocarcinoma was the main pathological type, accounting for about 2/3 of all patients, which may be due to a slightly higher percentage of female patients. Without any adjuvant therapies, the patients with either adenocarcinoma or squamous carcinoma did not show significant difference in survival. Patients with invasion of the pleura accounted for about 2/3 of all patients, and those with vascular invasion accounted for about 1/3. Both factors had a significant impact on prognosis.

Tumor size and lymph node status are important prognostic factors for lung cancer [11–14]. For example, for the development of T staging criteria, from T1a to T2b, the difference between stages is 1 cm, showing that the criteria of staging is to a large extent very refined, which leaves little room for further improvement. We can see that using such fine T-staging criteria, prognosis of patients with different T stages can be still distinguished. Thus, T staging has a significant impact on the prognosis of lung cancer. It is rare that patients with T3 tumors have no lymph node metastasis, whereas with T1a tumors, very few patients show the presence of metastatic lymph nodes. Therefore, in our study, no patients with stage IIB tumors were at stage T3N1M0 or T1aN1M0. From Table 1, we can clearly see that in the eighth edition of lung cancer TNM staging criteria, T1b corresponds to T1a of the seventh edition, T1c corresponds to T1b of the seventh edition, while the T2a stage of the seventh edition is divided into T2a and T2b in the eighth edition according to whether the tumor is larger than 3 cm but no larger than 4 cm and larger than 4 cm but no larger than 5 cm, respectively. In this case, under N1 stage, combining patients with T1b, T1c, T2a, or T2b four different T stages into IIB stage may result in mixing of different survivals.

We first analyzed the survival of patients in the four groups: T1b, T1c, T2a, and T2b. The p value of overall difference is 0.005, indicating a significant difference in survival, at least between two stages. Therefore, we compared the survivals between the two adjacent stages. The survival curves of the three pairs of comparison -T1b vs. T1c, T1c vs. T2a, and T2a vs. T2b are shown in Figure 2, with no significant differences in any of the three comparisons. Thus, under N1 stage, the degree of difference between adjacent T stages is reduced.

Thus, we again compared survival differences between non-adjacent T stages. The survival curves of the three comparisons -T1b vs. T2a, T1b vs. T2b, and T1c vs. T2b are shown in Figure 3, showing significant differences for all three comparisons, with p values are all less than 0.05. Therefore, subgroups of patients with different prognosis still exist among stage IIB patients.

In precision medicine, different therapeutic regimens should be used specifically for patients with different prognostic features. This requires us to standardize the treatment process, strengthen clinical research, and conduct randomized controlled clinical trials as soon as possible to obtain reliable large disease-related data, providing basis for the initiation, development and prognosis of diseases, as well as the analysis of drug resistance.

In conclusion, subclassification of IIB stage lung cancers is of prominent prognostic significance. In the future, more clinical randomized controlled trials are required to complete the data, in order to more accurately predict the prognosis of patients.

## MATERIALS AND METHODS

### Ethics statement

Investigation has been conducted in accordance with the ethical standards and according to the Declaration of Helsinki and according to national and international guidelines and has been approved by the authors' institutional review board. Informed consent has been obtained.

### Patients

In this retrospective study, 226 cases of IIB stage tumors were selected from a total of 1318 lung cancer patients treated without adjuvant therapies, who underwent radical surgery at the Liaoning Cancer Hospital and Institute between 2001 and 2010. Of the study group, 23 (10.2%) patients had pT1b, 30 (13.3%) patients had pT1c, 118 (52.2%) patients had pT2a, and 55 (24.3%) had pT2b cancers. The selection criteria for inclusion were as follows: (1) curative operations were performed; (2) the resected specimens were pathologically examined; (3) no adjuvant therapies were accepted; and (4) the patient medical records were complete. In practice, more than two pathologists were involved in corroborating the histological diagnosis after operation for all the cases.

### Classifications and subclassifications of stage IIB lung cancer

In the eighth edition of TNM staging criteria for lung cancer, stage IIB includes T3N0, T1 (T1a, T1b, T1c) N1, and T2 (T2a, T2b) N1. In our study, there were no patients at T3N0 or T1aN1 stage. Therefore, our patients were all at N1 stage, and according to the T staging, they were divided into four categories: T1b, T1c, T2a, and T2b.

### Statistical analysis

Data from all eligible patients were analyzed for overall survival. All data were analyzed using SPSS statistics software (Version 17.0). Overall survival was analyzed using the Kaplan–Meier method. The log-rank test was used to analyze survival differences. Two-sided p values were calculated for all tests and are reported here. A p value less than 0.05 was considered to indicate statistical significance.

## Abbreviations

AJCC/UICC: American Joint Committee on Cancer/Union for International Cancer Control
TNM: tumor-node-metastasis
